# Effect of SIS3 on Extracellular Matrix Remodeling and Repair in a Lipopolysaccharide-Induced ARDS Rat Model

**DOI:** 10.1155/2020/6644687

**Published:** 2020-11-25

**Authors:** Qiong Liang, Qiqing Lin, Yueyong Li, Weigui Luo, Xia Huang, Yujie Jiang, Chunyan Qin, Jin Nong, Xiang Chen, Suren Rao Sooranna, Liao Pinhu

**Affiliations:** ^1^The First Clinical Medical College of Jinan University, Guangzhou City, Guangdong Province, China; ^2^Department of Respiratory Medicine, Affiliated Hospital of Youjiang Medical University for Nationalities, Baise City, Guangxi Province, China; ^3^Emergency Department, Affiliated Hospital of Youjiang Medical University for Nationalities, Baise City, Guangxi Province, China; ^4^Department of Intervention Medicine, Affiliated Hospital of Youjiang Medical University for Nationalities, Baise City, Guangxi Province, China; ^5^Intensive Care Unit, Affiliated Hospital of Youjiang Medical University for Nationalities, Baise City, Guangxi Province, China; ^6^Intensive Care Unit, People's Hospital of Guangxi Zhuang Autonomous Region, Nanning City, Guangxi Province, China; ^7^Department of Metabolism, Digestion and Reproduction, Imperial College London, Chelsea & Westminster Hospital, 369 Fulham Road London, SW10 9NH, UK

## Abstract

The remodeling of the extracellular matrix (ECM) in the parenchyma plays an important role in the development of acute respiratory distress syndrome (ARDS), a disease characterized by lung injury. Although it is clear that TGF-*β*1 can modulate the expression of the extracellular matrix (ECM) through intracellular signaling molecules such as Smad3, its role as a therapeutic target against ARDS remains unknown. In this study, a rat model was established to mimic ARDS via intratracheal instillation of lipopolysaccharide (LPS). A selective inhibitor of Smad3 (SIS3) was intraperitoneally injected into the disease model, while phosphate-buffered saline (PBS) was used in the control group. Animal tissues were then evaluated using histological analysis, immunohistochemistry, RT-qPCR, ELISA, and western blotting. LPS was found to stimulate the expression of RAGE, TGF-*β*1, MMP2, and MMP9 in the rat model. Moreover, treatment with SIS3 was observed to reverse the expression of these molecules. In addition, pretreatment with SIS3 was shown to partially inhibit the phosphorylation of Smad3 and alleviate symptoms including lung injury and pulmonary edema. These findings indicate that SIS3, or the blocking of TGF-*β*/Smad3 pathways, could influence remodeling of the ECM and this may serve as a therapeutic strategy against ARDS.

## 1. Introduction

Acute respiratory distress syndrome (ARDS) is a diffuse pulmonary parenchymal injury characterized by injury of the pulmonary epithelium and vascular endothelial cells as well as inflammatory infiltration, hyaline membrane formation, matrix repair, and pulmonary interstitial fibrosis. Lipopolysaccharide (LPS) is a major component of the cell walls of gram-negative bacteria that possesses powerful inflammatory effects and plays a significant role in the occurrence and progression of ARDS [1]. The pathogenesis of ARDS is complex and is a hotspot in the study of pulmonary diseases [2, 3].

Extracellular matrix (ECM) metabolism serves in the regulation of lung function and morphology. ECM remodeling encompasses changes in fibrin collagen, the basement membrane, and elastin. Matrix cells synthesize and secrete extracellular polysaccharides and macromolecules, such as proteins and proteoglycans, which are distributed on the cell surface or between cells, forming a network structure between cells. During the course of ARDS, activated polymorphonuclear leukocytes (PMNs) and macrophages release various inflammatory cytokines and proteolytic enzymes that degrade the ECM, affecting and changing the functions of the cell and matrix and triggering ECM remodeling. At the same time, proteolytic enzymes released by the PMNs and macrophages can digest alloproteins and damage host proteins, including the ECM.

Proteolytic enzymes also support and establish connections as well as affecting various pathophysiological processes, such as cell shape, metabolism, function, adhesion, proliferation, and differentiation through the cell signaling transduction system [3]. The temporary pulmonary matrix formed following ARDS injury can activate an inflammatory reaction and induce the transformation of epithelial cells into fibroblasts, leading to local connective tissue and continuous matrix remodeling during the repair of lung injury. Intra-alveolar fibrosis (remodeling) can cause alveolar occlusions and eventually a loss of alveolar function [4].

Persistent inflammatory and fibrotic alveolitis and increased inflammation in the early stages of ARDS are the primary mechanisms which lead to alveolar membrane and fibrosis injuries. The resulting persistent inflammatory response can destroy the ECM, leading to changes in a lung structure. Some studies have shown that changes in the fibroplasia in ARDS may be related to persistent inflammation following lung injury, the generation and degradation of the temporary matrix after lung injury, and the change in the lining components of the epithelial cells [5]. According to the extent of ECM remodeling, the injured lung may be completely repaired. However, ECM remodeling disorders can lead to pulmonary fibrosis (PF); therefore, the prognosis for ARDS can be very poor [6].

Receptor for advanced glycation end-products (RAGE), matrix metalloproteinases (MMPs), and tissue inhibitors of metalloproteinases (TIMPs) participate in remodeling of the ECM in ARDS [4]. RAGE is also one of the biomarkers of pulmonary epithelial injury [7, 8] and is a transmembrane and multiligand receptor belonging to the immunoglobulin superfamily. The receptor structure includes three parts: extramembrane, transmembrane, and intracellular membrane domains. RAGE is primarily distributed in type I alveolar epithelial cells, alveolar macrophages, and bronchiole epithelial cells, and it is highly expressed in the lung but low in other organs of the body. RAGE also plays a protective role in the lung, where its activation modulates cell signaling and propagation of the inflammatory response [9]. Overexpression of RAGE can increase alveolar cell apoptosis and inhibit cell proliferation [10]. Moreover, the absence of RAGE in pulmonary epithelial cells can lead to their fibrosis which can, in turn, lead to the occurrence and development of PF [11]. Calfee et al. [12] reported an increase in RAGE plasma levels in patients with severe ARDS, and a relationship between mortality in ARDS patients with high tidal volume ventilation was further established.

MMPs usually play important roles in various pathological conditions, such as wound repair and inflammatory leukocyte infiltration. In destructive lung diseases, it has been reported that MMP1, MMP2, MMP3, MMP7, MMP8, MMP9, MMP10, and MMP12 contribute to tissue injury [13]. However, in acute lung injury, especially in LPS-induced ARDS, the activation of MMP2 and MMP9 exacerbates pulmonary inflammation [14]. MMP2 can degrade a variety of collagen substrates, and it stimulates the release of vascular endothelial growth factor, thus driving endothelial genesis, and contributes to tissue repair and/or damage, while MMP9 degrades the ECM components such as gelatin, fibronectin, and fibrillin. Uncontrolled synthesis and degradation of the ECM play an important role in various pathological conditions. The pathogenesis of ARDS can lead to expression of MMPs in cells including alveolar epithelial cells, macrophages, neutrophils, and fibroblasts [3, 5, 15]. MMPs are zinc-dependent endopeptidases that are upregulated in most pulmonary inflammatory diseases, which lead to the degradation of the pulmonary ECM [16]. MMPs primarily affect the biological activities of these mediators by degrading the ECM and participating in the lysis of inflammatory cytokines and chemokines secreted into the ECM, thus influencing cell behavior or aggravating lung injury [17, 18]. MMPs, in turn, regulate inflammation by interacting with cytokines and chemokines [19].

The prognosis is affected by cell proliferation and fibrosis-induced PF after ARDS. Many studies have shown that transforming growth factor- (TGF-) *β* is related to the repair of posttraumatic fibrosis. TGF-*β* is produced by monocytes, lymphocytes, epithelial cells, and fibroblasts, and these regulate the proliferation, differentiation, migration, adhesion, and metabolism of the ECM through autocrine or paracrine mechanisms while participating in embryonic development, injury, and repair [20]. TGF-*β* is highly expressed in injured cells and tissues and is involved in the repair of fibrosis and proliferation of various injuries including ARDS [21, 22]. The longer the duration of highly expressed TGF-*β*, the more difficult it is to alleviate the clinical manifestations of ARDS. Clinical studies have shown that high levels of TGF-*β* were associated with more serious cases of ARDS and those with worse prognosis [23].

The most important role of TGF-*β*1 in the pathology of PF is the expression of the ECM gene mediated by the molecules referred to as small mothers against decapentaplegic 3 (Smad3). In canonical TGF-*β*/Smad signaling, the active TGF-*β* ligand binds to the receptor, TbRII, which then recruits TbRI. TGF-*β* subsequently triggers a cell response by activating the TGF-*β* receptor, which triggers the phosphorylation of Smad2 and Smad3 proteins. Phosphorylated Smad2 or 3 can bind with common-mediator Smad (co-Smad) and is translocated into the nucleus to exert its transcriptional activity in order to regulate downstream gene expression [24]. Smad3, unlike Smad2, has been shown to play a profibrotic role in kidney fibrosis [25]. Smad3 directly regulates the expression of various fibrogenic genes, such as those associated with the ECM and TIMPs [26]. Furthermore, Smad3 can also stimulate TGF-*β*1 expression to enhance TGF-*β*/Smad signaling through positive feedback. The prognosis is thereby influenced by cell proliferation following ARDS. Selective inhibitors of Smad3 (SIS3) are synthetic chemicals which strongly inhibit the phosphorylation of Smad3 induced by TGF-*β*1 [27]. Recent studies have shown that SIS3 has antifibrotic and anti-inflammatory effects in bleomycin- (BLM-) induced PF in mice [28]. Evidently, SIS3 attenuates the regulatory mechanism of TGF-*β* by selectively inhibiting Smad3 [28].

These data illustrate the antifibrotic and anti-inflammatory effects of SIS3 *in vivo*. However, its role in the pathogenesis of ARDS requires further elucidation. It is anticipated that studies into matrix remodeling and repair in ARDS may provide potential intervention indicators for improving the prognosis of ARDS [2, 3].

## 2. Materials and Methods

### 2.1. LPS-Induced ARDS Rat Model and Experimental Program

All procedures in this study were conducted in accordance with the guidelines of the National Institute of Health and approved by the Animal Protection and Utilization Committee of the Youjiang Medical University for Nationalities. Six- to eight-week-old male Sprague-Dawley (SD) rats were obtained from the Laboratory Animal Center of the Youjiang Medical University for Nationalities (Baise, Guangxi Province, China) and raised in a pathogen-free environment. A total of 40 adult male SD rats were randomly divided into four groups: a control group (CTL, *n* = 10, no treatment); a ARDS group (ARDS, *n* = 10, intratracheal instillation of 5 mg/kg of LPS, Sigma-Aldrich, St. Louis, MO, USA); and a ARDS and SIS3 group (ARDS + SIS3, *n* = 10, intraperitoneal injection of 2.5 mg/kg of SIS3 [29] for 2 h following 5 mg/kg of LPS intratracheal instillation). The ARDS and phosphate-buffered saline (PBS) group (ARDS + PBS, *n* = 10), the same as in the ARDS + SIS3 group but where an equal amount of PBS replaced the SIS3.

The rats used in this study were anesthetized 24 h after LPS intratracheal instillation, with ketamine at a dose of 80 mg/kg, together with xylazine at a dose of 8 mg/kg, by intraperitoneal injection, as recommended by the IACUC guidelines. 5-8 mL blood biopsies were collected by cardiac puncture under deep terminal anaesthesia with 25 G needled syringes inserted into the left ventricle. Blood biopsies were withdrawn slowly to avoid the heart from collapsing. After taking blood biopsies, the rats were killed by cervical dislocation, and then, the lung tissues were collected.

Following 30 min of coagulation, tubes were centrifuged at 2000×g for 20 min and stored at −80°C for subsequent analysis. The bronchoalveolar lavage fluid (BALF) of the left lung was obtained using endotracheal intubation 24 h after LPS intratracheal instillation, and the lung was then washed with PBS (4°C, 15 mL/kg) five times. The flushing fluid was centrifuged at 4°C for 10 min at 1500×g, and the supernatant was collected for protein concentration measurements. The sediment was resuspended using 50 *μ*L of PBS, and the number of cells was counted. The right upper lobe was examined via histopathology and immunohistochemistry, and the right part of the middle lobe was weighed to obtain the wet-to-dry (*W*/*D*) weight ratio. The rest of the lower lobe of the right lung was preserved at −80°C, and the total RNA and/or protein was isolated. For histopathology and immunohistochemical staining, tissues were fixed in 10% neutral formaldehyde and paraffin embedded, and 4 *μ*m thickness sections were cut. These were either stained with hematoxylin and eosin (HE) or subjected to immunohistochemical staining.

### 2.2. Masson's Trichrome Staining

A commercial Masson's trichrome staining kit (Solarbio, #G135) was used to perform Masson's staining, considering collagen is the main part of ECM [30]. Briefly, lung biopsies were embedded in paraffin after fixation with 4% buffered paraformaldehyde, and sectioned into 4 *μ*m. After dipping in to Bouin's solution for 2 h at 37°C, the slides were stained with Celestine blue dye for 2 min and Mayer's solution for 3 min. Following an acid ethanol differentiation step for 10 sec, sections were dipped in Lichun red magenta solution for 10 min, phosphomolybdic acid solution for 10 min, aniline blue solution for 5 min, and a weak acid for 2 min. The slides were carefully washed with PBS after each staining procedure. Then, the slides were dipped twice in ethanol for 10 sec each. After two dips of 2 min in xylene, the slides were mounted with Balsam.

### 2.3. RT-qPCR

Gene expressions for RAGE, TGF-*β*1, MMP2, and MMP9 mRNAs from rat lung tissue were evaluated using the FastStart Universal SYBR Green Master (ROX) (Roche, Germany) and real-time PCR. The RNA of the frozen lung tissue was isolated using a total RNA rapid separation kit (Tiangen, China), and the DNA strand was synthesized by using the first-strand cDNA synthesis kit (Thermo Scientific k1622, USA) according to the manufacturer's instructions. All primers used were synthesized by Shenggong (Shanghai, China; Table 1). Following 5 min of initial activation at 95°C, PCR was carried out for 40 cycles at 95°C for 10 s and 60°C for 30 s. Glyceraldehyde-3-phosphate dehydrogenase (GAPDH) was measured simultaneously and used as the housekeeping gene. The threshold cycle (Ct) values were measured as previously described, and the comparative gene expression was calculated using the 2^-*ΔΔ*Ct^ method.

### 2.4. ELISA

RAGE, TGF-*β*1, MMP2, and MMP9 concentrations in the rat sera and BALF were determined using a rat RAGE ELISA kit (EK0971 from Boster Biological Technology, China), a rat TGF-*β*1 ELISA kit (MB100B, R&D Systems), a rat MMP2 ELISA kit (MMP200, R&D Systems), and a rat MMP9 ELISA kit (CSB-E08008r Cusabio, Wuhan, China), respectively, following the manufacturer's protocols.

### 2.5. Lung *W*/*D* Ratio Analysis

The ARDS rats were euthanized after 24 h, and the right middle lobe was dissected and weighed. Subsequently, the tissues were dried in an oven at 60°C for 72 h and reweighed. The corresponding *W*/*D* weight ratios were then calculated.

### 2.6. Neutrophil Number

The total cell number and neutrophils in the BALF were counted using a hemocytometer and light microscopy, and the ratio of neutrophils to total cells was calculated.

### 2.7. Western Blot Analysis

The protein concentration of lung homogenate was measured using bovine serum albumin (BSA) as the standard. Equivalent amounts of total protein (50 mg) were separated on 10–12% SDS-PAGE gels subjected to electrophoresis and were electrophoretically transferred onto nitrocellulose membranes. These were blocked with 5% milk for 30–60 min and incubated with primary antibodies against RAGE (1 : 500 dilution, Sc365154, Santa Cruz Biotechnology), TGF-*β*1 (1 : 1000 dilution, 3711, Cell Signaling Technology), MMP2 (1 : 1000 dilution, GTX104577, Gene Tex), MMP9 (1 : 1000 dilution, 13667, Cell Signaling Technology), Smad3 (1 : 1000 dilution, 9523, Cell Signaling Technology), phospho-Smad3 (1 : 1000 dilution, 9520, Cell Signaling Technology), and GAPDH (1 : 20000 dilution, 10494-1-AP, Proteintech Group) at 4°C overnight. Following primary antibody incubation, membranes were incubated with goat anti-rabbit/rat IgG (1 : 5000 dilution, 9936, Cell Signaling Technology) linked to horseradish peroxidase at room temperature for 60 min. The proteins recognized by the antibody complexes were then visualized using enhanced chemiluminescence (Solarbio, Beijing, China). The optical density of each marker band was analyzed using ImageJ version 1.48 software.

### 2.8. Immunohistochemistry

4 *μ*m thickness paraffin sections were pasted onto slides for conventional dewaxing treatment. The sections were microwaved in a citrate buffer for 15 min (pH 6.0), and endogenous peroxidase was blocked by hydrogen peroxide and goat serum. The slices were then incubated with polyclonal RAGE (1 : 200, Sc365154, Santa Cruz Biotechnology, United Kingdom), TGF-*β*1 (1 : 100, BS6152, Bioword Technology, Inc., China), MMP2 (1 : 200, GTX104577, GeneTex, North, America), and MMP9 (1 : 50, BM4089, Boster Biological Technology, China) antibodies overnight at 4°C. The equivalent concentrations of polyclonal nonimmune IgG were used as controls. Then, sections were incubated with secondary HRP-En Vision IgG antibody at room temperature for 20 min and followed by incubation with a streptavidin solution. Color development was carried out using 3,3-diaminobenzidine tetrahydrochloride.

### 2.9. HE Staining and Lung Injury Score

The lung tissues were immersed in 4% paraformaldehyde for 24 h and transferred to ethanol, dehydrated through a serial alcohol gradient, and embedded in paraffin wax blocks. 4 *μ*m thickness lung tissue sections were dewaxed in xylene, which then underwent HE staining. Lung injury was scored according to the following five categories, which were assessed using a rating of 0–2 for each of the following standards: (a) neutrophils in the alveolar space, (b) neutrophils in the interstitial space, (c) hyaline membranes, (d) protein fragments filled with air spaces, and (e) thickening of the alveolar septum. The total damage score was calculated by the following equation: score = (20 × *a* + 14 × *b* + 7 × *c* + 7 × *d* + 2 × *e*)/(number of fields × 100) [31].

### 2.10. Statistical Analysis

The data are expressed as the means ± standard deviations (SD). The statistical analysis was conducted using SPSS 19.0 software and GraphPad Prism 5.0 (San Diego, California, USA). The comparisons between two groups were analyzed using Student's *t*-test. The differences among multiple groups were analyzed using ANOVA. In all cases, *P* < 0.05 was considered to be statistically significant.

## 3. Results

### 3.1. LPS-Induced RAGE, TGF-*β*1, MMP2, and MMP9 Genes Were Suppressed by SIS3 in the ARDS Rat Model

**Figure 1:**
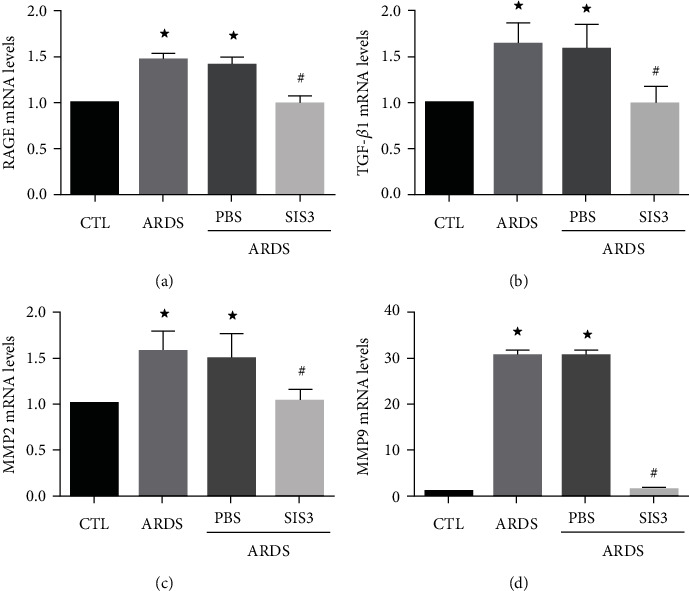
SIS3 inhibits mRNA expression of RAGE, TGF-*β*1, MMP2, and MMP9 in lung homogenates of ARDS rats. The mRNA levels of RAGE, TGF-*β*1, MMP2, and MMP9 were determined using real-time PCR and were standardized to *β*-actin. The results suggested that SIS3 inhibited the increase of (a) RAGE, (b) TGF-*β*1, (c) MMP2, and (d) MMP9 in LPS-induced lung homogenates of ARDS rats, while no effect was seen upon pretreatment with PBS. The data are presented as the means ± SD (*n* = 10). ^★^*P* < 0.05 vs. CTL; ^#^*P* < 0.05 vs. ARDS.

The expression of RAGE, TGF-*β*1, MMP2, and MMP9 mRNA in lung tissues was analyzed using RT-qPCR in the ARDS rat model. Accordingly, RAGE, TGF-*β*1, MMP2, and MMP9 mRNA expression was found to be increased in lung homogenates after LPS induction. Meanwhile, the expression of RAGE, TGF-*β*1, MMP2, and MMP9 mRNA in lung homogenates was observed to be suppressed by SIS3 (*P* < 0.05 in all cases; Figures 1(a)–1(d)).

### 3.2. SIS3 Inhibited LPS-Induced RAGE, TGF-*β*1, MMP2, and MMP9 Protein Expression in ARDS Rats

**Figure 2:**
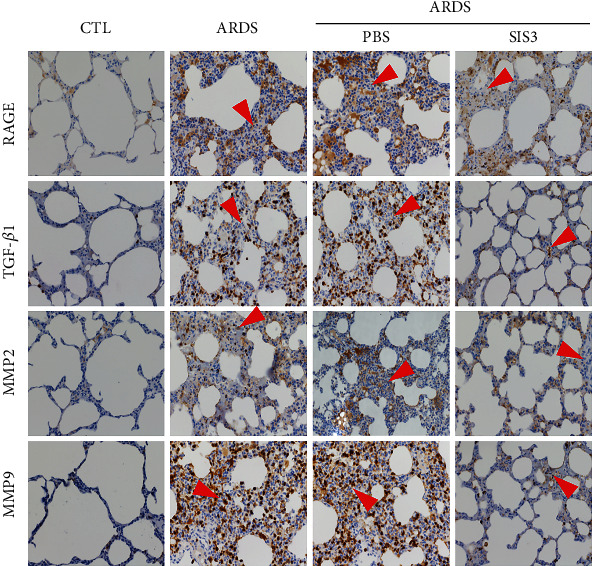
SIS3 pretreatment decreases the protein expression and localization of RAGE, TGF-*β*1, MMP2, and MMP9 in ARDS rats. Immunohistochemical staining of lung tissue sections showed that RAGE, TGF-*β*1, MMP2, and MMP9 were expressed in the bronchial smooth muscle, airways, and alveolar epithelial cells of rats. The expression levels of RAGE, TGF-*β*1, MMP2, and MMP9 in the ECM of ARDS rats induced by LPS were significantly higher than those of the control group. RAGE, TGF-*β*1, MMP2, and MMP9 were reduced by pretreatment with SIS3, and no effect was found upon pretreatment with PBS. The micrographs were magnified at 400x. The red triangles indicate infiltrated leukocytes.

Immunohistochemical staining of lung tissue sections showed that RAGE, TGF-*β*1, MMP2, and MMP9 were primarily expressed in the cytoplasm of alveolar epithelial cells in normal lung tissues of rats. RAGE, TGF-*β*1, MMP2, and MMP9 expression in the ARDS group was observed to be significantly higher than those in the control group. Notably, treatment with SIS3 decreased RAGE, TGF-*β*1, MMP2, and MMP9 expression. However, PBS did not affect RAGE, TGF-*β*1, MMP2, and MMP9 expression in the ARDS + PBS group of rats (Figure 2).

### 3.3. SIS3 Relieved Acute Lung Inflammation and Injury in the ARDS Rat Model

**Figure 3:**
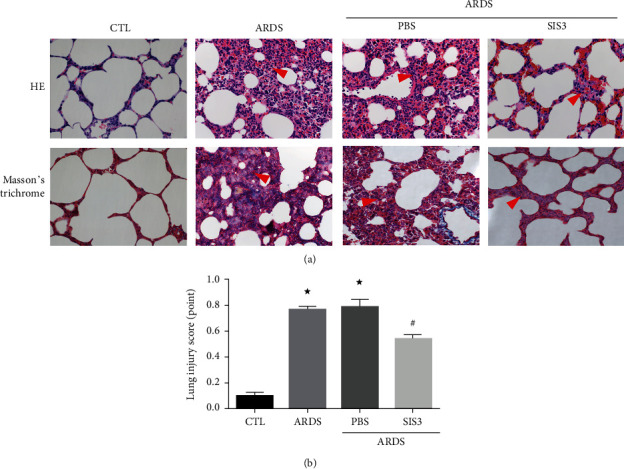
The inflammatory response was reduced by SIS3 pretreatment in LPS-induced acute lung injury. (a) Histological staining was performed to compare lung injury among the different groups. Both HE staining (upper panel) and Masson's trichrome staining (lower panel) show drastic leukocyte infiltration in the lung interstitium in the ARDS group, and the leukocyte infiltration was markedly alleviated after treatment of SIS3. Masson's trichrome staining reveals abnormal fibrosis and collagen distribution which was stained as blue, in the ARDS group, and significantly less blue stain is seen in the ARDS group treated with SIS3. The red triangles indicate the infiltrated sites. The photomicrographs were magnified to 400x. (b) HE staining was used to score lung injury. In the ARDS group, the lung injury was 0.77 ± 0.01, and after treatment with SIS3, the score decreased to 0.54 ± 0.01 (^★^*P* < 0.05 vs. CTL; ^#^*P* < 0.05 vs. ARDS). Data are presented as the means ± SD (*n* = 10).

HE staining of pathological rat lung tissues demonstrated the presence of clear lung structures, with little or no alveolar and alveolar interstitial inflammatory cell infiltration, no edema, and no erythrocytes or other exudates in the control group. In the ARDS group, lesions were identified to be extensive and primarily manifested as lung edema, hemorrhage, infiltration of neutrophils, alveolar damage, pulmonary interstitial thickening, narrowing of the alveolar cavity, and an increasing lung injury score. The pathological changes in the ARDS + SIS3 group were evident by slight edema with a small amount of spotting and sheet hemorrhaging. Lung injury and inflammation (usually characterized by leukocyte infiltration) in the rats were alleviated by the inhibitor of SIS3 (Figure 3(a)). Moreover, the lung injury scores decreased from 0.77 ± 0.01 to 0.54 ± 0.01 (Figure 3(b)), whereas pretreatment with PBS had no effect in ARDS rats.

**Figure 4:**
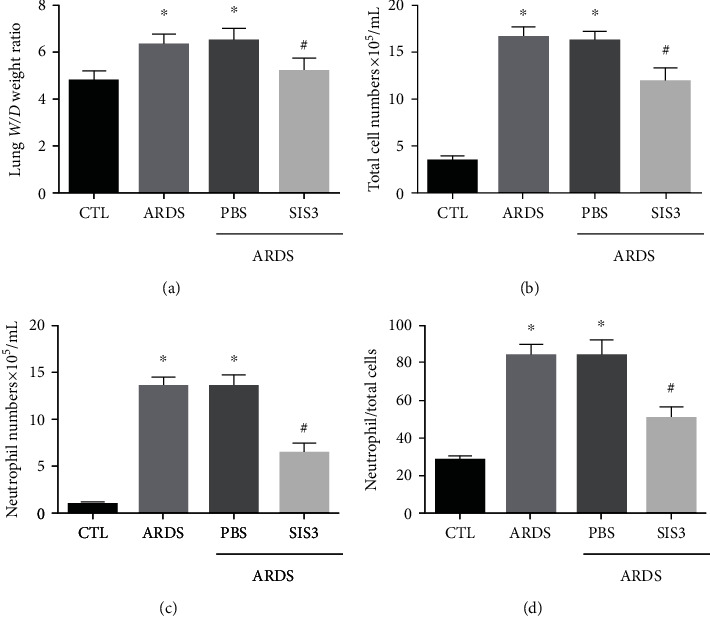
SIS3 pretreatment alleviates lung injury and focal inflammation in LPS-induced ARDS. (a) BALF was collected 24 h after intratracheal instillation of LPS. The alternation of the alveolar capillary barrier was weakened by SIS3 pretreatment in ARDS rats, which was seen in an increase of the lung *W*/*D* ratio. (b–d) The total cells, neutrophils, and neutrophil ratio in the BALF were higher in the ARDS group than in the control group. The values were also lower in the ARDS + SIS3 group when compared to the ARDS group, respectively. The data are presented as the means ± SD (*n* = 10). ^★^*P* < 0.05 vs. CTL; ^#^*P* < 0.05 vs. ARDS.

Rats administered with LPS exhibited an altered alveolar capillary barrier with increased lung *W*/*D* weight ratios (Figure 4(a)), upregulated inflammatory responses with an increased number of cells (Figure 4(b)), increased number of neutrophils (Figure 4(c)), and increased neutrophil ratios (Figure 4(d)) in the BALF. As illustrated in Figure 4(a), the *W*/*D* ratio of the ARDS group was observed to be 33.7 ± 4.7% which was higher than that of the control group, indicating the presence of pulmonary edema (*P* < 0.05, *n* = 10). However, the *W*/*D* ratio in the ARDS + SIS3 group was 18.3 ± 2.15%, which was lower than that in the ARDS group, indicating that SIS3 attenuated the degree of pulmonary edema induced by LPS (*P* < 0.05 in all cases).

The total number of cells in the BALF was markedly higher in the ARDS group than the control group (control group: 3.31 ± 0.2 × 10^5^/mL, ARDS group: 16.54 ± 0.33 × 10^5^/mL; *P* < 0.05 in all cases). Additionally, the total number of cells in the BALF decreased by 31.2 ± 1.7% in the ARDS+SIS3 group when compared to the ARDS group. The number of neutrophils was observed to be higher in the ARDS group than in the control group (control group: 0.93 ± 0.07 × 10^5^/mL, ARDS group: 13.55 ± 0.29 × 10^5^/mL; *P* < 0.05). The number of neutrophils also decreased by 50.7 ± 2.35% in the ARDS + SIS3 group when compared to the ARDS group (*P* < 0.05 in all cases). The changes in the neutrophil ratios were found to be consistent with changes in the total number of cells as well as the number of neutrophils.

### 3.4. The Effect of SIS3 on the ECM Expression due to LPS-Induced ARDS Rats

**Figure 5:**
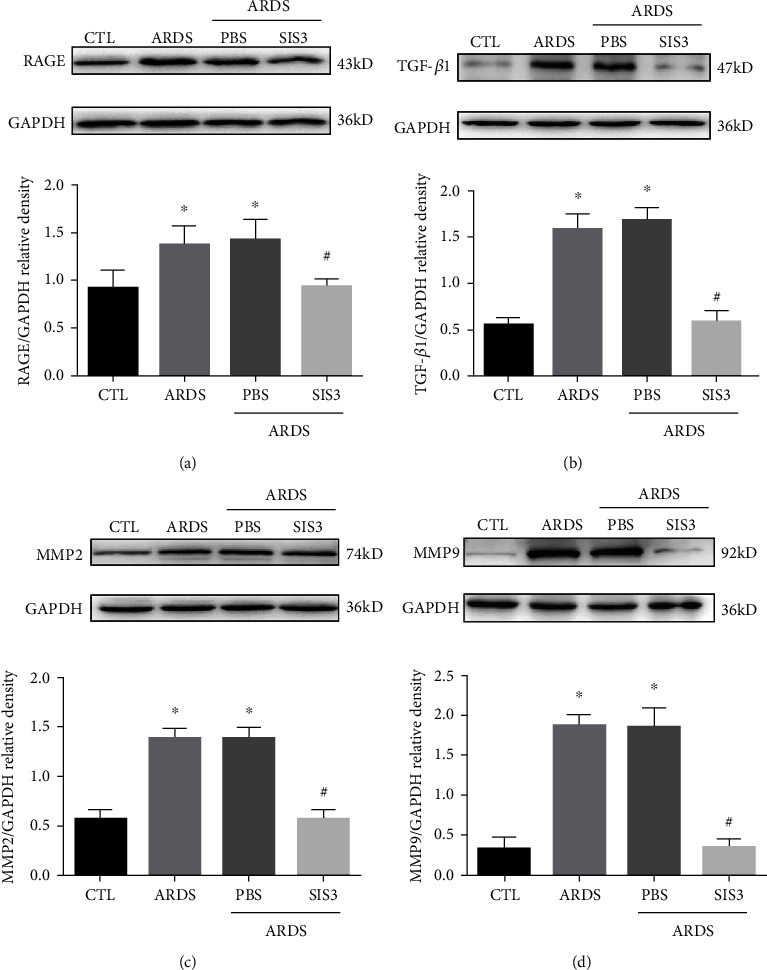
SIS3 inhibited the protein expression of RAGE, TGF-*β*1, MMP2, and MMP9 proteins in lung homogenates acquired from ARDS rats. The expression levels of RAGE, TGF-*β*1, MMP2, and MMP9 proteins in lung tissue homogenates, sera, and BALF of ARDS rats and were determined using western blotting analysis at 24 h after LPS intervention. The results of western blotting showed that SIS3 inhibited LPS-induced (a) RAGE, (b) TGF-*β*1, (c) MMP2, and (d) and MMP9 protein expression in the lung homogenates. The data are presented as the means ± SD (*n* = 10). ^★^*P* < 0.05 vs. CTL; ^#^*P* < 0.05 vs. ARDS.

SIS3 pretreatment reduced inflammation in the ARDS rat model. Western blot analysis of homogenized rat lung tissue showed that, compared to those of the control group, the protein levels of RAGE (Figure 5(a)), TGF-*β*1 (Figure 5(b)), MMP2 (Figure 5(c)), and MMP9 (Figure 5(d)) in the lung tissues of the ARDS group increased by 1.54 ± 0.05, 2.87 ± 0.07, 2.44 ± 0.12, and 6.38 ± 1.27 times, respectively, while those of the ARDS + SIS3 group decreased by 19.34 ± 1.74, 63.10 ± 1.89, 57.90 ± 4.97, and 81.78 ± 2.09% compared to that of the ARDS group, respectively (*P* < 0.05 in all cases). The levels of RAGE, TGF-*β*1, MMP2, and MMP9 in the ARDS+SIS3 group were 19.34 ± 1.74, 63.10 ± 1.89, 57.90 ± 4.97, and 81.78 ± 4.97% lower than those in the ARDS group, respectively.

Immunohistochemistry measurements of the lung sections showed markedly increased leukocyte infiltration in the ARDS group, and considering that ARDS is an acute inflammatory disease, most of these should be neutrophils [32] (Figures 2 and 3). Neutrophils play an important role in host defense against infection through the secretion of various factors such as proteinases and neutrophil extracellular traps. However, these factors can also be toxic which will therefore result in tissue damage [33]. After treatment with SIS3, the infiltrated leukocytes were markedly decreased (Figures 2 and 3).

**Figure 6:**
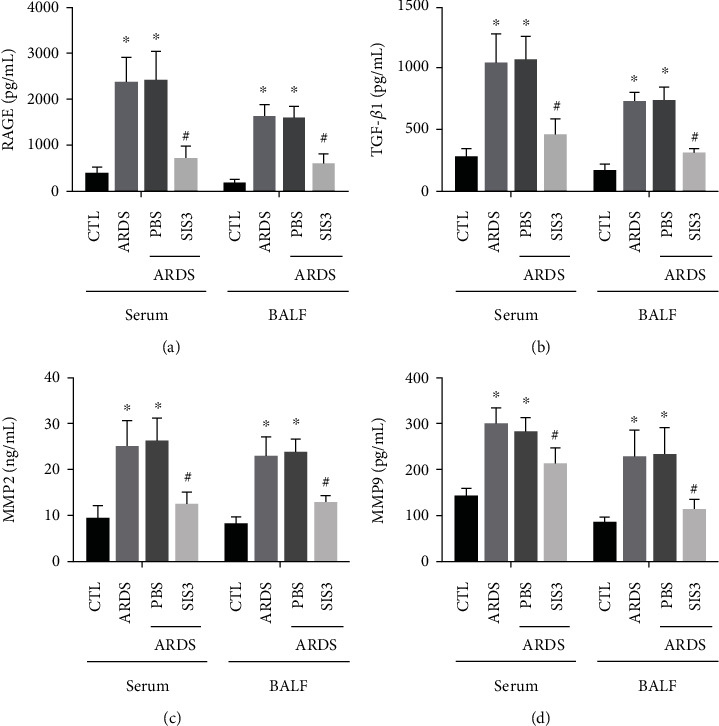
SIS3 pretreatment prevented the reduction of the expression of RAGE, TGF-*β*1, MMP2, and MMP9 in LPS-induced ARDS. The effects of SIS3 on the protein expression levels of (a) RAGE, (b) TGF-*β*1, (c) MMP2, and (d) MMP9 in the BALF and sera of the ARDS rats were determined using ELISA. The results demonstrated that the levels of RAGE, TGF-*β*1, MMP2, and MMP9 proteins were significantly higher in the ARDS group than in the control group, while the levels of RAGE, TGF-*β*1, MMP2, and MMP9 in the SIS3 group were lower than those in the ARDS group. The protein levels of RAGE, TGF-*β*1, MMP2, and MMP9 in LPS-administered pretreatment with PBS were not different from those of the ARDS group. The data are presented as the means ± SD of three independent experiments in triplicate (*n* = 10). ^★^*P* < 0.05 vs. CTL; ^#^*P* < 0.05 vs. ARDS.

In addition, ELISA demonstrated that the serum and BALF levels of RAGE, TGF-*β*1, MMP2, and MMP9 in the ARDS group were 8.02 ± 1.7, 4.16 ± 0.49, 2.87 ± 0.29, and 2.13 ± 0.12 times (for sera) and 10.70 ± 1.36, 5.38 ± 0.87, 2.93 ± 0.24, and 2.76 ± 0.24 times (for BALF) higher than those in the control group (*P* < 0.05 in all cases). The levels of RAGE, TGF-*β*1, MMP2, and MMP9 in the ARDS + SIS3 group were 68 ± 4.98, 55.20 ± 4.71, 49.20 ± 3.90, and 28.40 ± 4.48% (for sera) and 64.30 ± 4.37, 58.80 ± 3.04, 42.70 ± 3.49, and 47.40 ± 6.38% (for BALF), which were lower than those in the ARDS group (*P* < 0.05 in all cases, Figures 6(a)–6(d)). The protein levels of RAGE, TGF-*β*1, MMP2, and MMP9 in rats pretreated with PBS were not significantly different from those seen in the ARDS group. In summary, SIS3 inhibited the RAGE, TGF-*β*1, MMP2, and MMP9 protein expression levels in both the serum and BALF of ARDS rats.

### 3.5. SIS3 Inhibited the Activation of Phospho-Smad3 in the ARDS Rat Model

**Figure 7:**
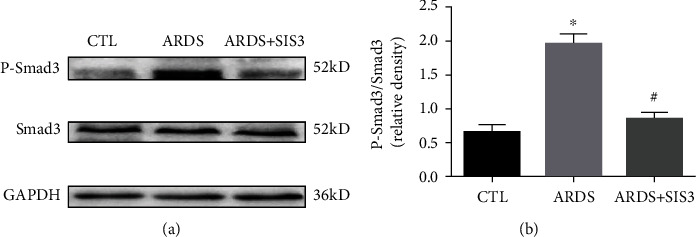
Phosphorylation of smad3 was inhibited by SIS3 in the lung tissue of ARDS rats. The activation of Smad3 was evaluated using western blotting analysis, and the amount of phospho-Smad3 formed was analyzed 24 h after treatment with either LPS or PBS. GAPDH was used as the loading control. The data are presented as the means ± SD (*n* = 10). ^★^*P* < 0.05 vs. CTL; ^#^*P* < 0.05 vs. ARDS.

Western blot analysis showed that LPS activated Smad3 signaling by increasing Smad3 phosphorylation, while SIS3 pretreatment blocked LPS-induced phospho-Smad3 (*P* < 0.05 in all case; Figure 7).

### 3.6. SIS3 Alleviated Abnormal Collagen Expression and Distribution

With Masson's trichrome staining, the fibrosis in different groups was compared and analyzed (Figure 3(a)). It was seen that pathological fibrosis was usually driven by the ECM. In this experiment, collagen fibers were shown in blue. In the ARDS group, markedly, more blue stain was seen in the lung interstitium which means there was an upregulated expression of collagen when compared with the CTL group. After treatment with SIS3, the abnormal collagen distribution was significantly alleviated, but this was not seen in the ARDS group treated with PBS.

## 4. Discussion

Acute lung injury (ALI) and ARDS are two types of pulmonary complications that are caused by various conditions, such as sepsis, trauma, and pulmonary inflammation. A key step in its development is an uncontrolled inflammatory response. In this regard, LPS has been shown to trigger the inflammatory response in a dose-dependent and cell-specific manner. Sepsis is the most important cause of ARDS [34]. LPS is a major component of gram-negative bacteria and has been used to induce ALI/ARDS and other inflammatory diseases in several *in vivo* experiments. Moreover, LPS has been successfully used in establishing an ARDS rat model [35]. Therefore, in this study, an LPS-induced ARDS rat model was established in our laboratory, and the remodeling role of the ECM gene, SIS3, in LPS-induced ARDS rats was analyzed. Accordingly, SIS3 treatment was found to regulate the secretion of cytokines as well as the degradation of collagen synthesis in remodeling and repairing the ECM, which reversed LPS-induced lung injury, improved pulmonary edema, improved histopathological features, reversed the protein and RNA expression of several ECM genes, downregulated the phosphorylation of Smad3, and repressed LPS-induced acute lung inflammation.

The ECM plays an important role in lung growth, development, lung tissue damage, and repair as well as being involved in the development of some lung diseases, significantly affecting the prognosis of ARDS. Li et al. [36] suggested that the inhibition of RAGE or NF-*κ*B could alleviate LPS-induced lung injury in neonatal rats. Additionally, accumulating data have suggested a crucial role of RAGE in the pathogenesis of ALI and ARDS, indicating that this receptor is potentially an important therapeutic participant in ALI/ARDS [10]. In an environment of advanced glycated end-products, the ECM is regulated by the RAGE-dependent pathway, which increases the expression of RAGE as well as MMP2 and MMP9 [37]. Both of these gelatinases degrade type IV collagen and are primary constituents of basement membranes and play an important role in lung pathology [38]. The action of gelatinase is essential for basement membrane remodeling, which is observed in various pulmonary inflammatory diseases such as ARDS. MMP primarily affects the biological activities of these mediators by degrading the ECM and participating in the lysis of inflammatory cytokines and chemokines secreted into the ECM, which ultimately influence cell behavior and aggravate lung injury [17, 18].

Torii et al. demonstrated a significantly higher concentration of MMP2 and MMP9 in the BALF of ARDS patients compared to healthy subjects [39]. The levels of these MMPs were found to be elevated in BALF of ALI patients [40]. LPS stimulation was shown to immediately activate MMP2 and MMP9 production in BALF among ALI rats [41]. In this study, LPS was found to increase RAGE, TGF-*β*1, MMP2, and MMP9 mRNA and protein expression in LPS-induced ARDS rats. These results were in accord with those of previous studies, which primarily implicated MMPs in the pathogenesis of ARDS in promoting lung inflammation and/or injury to the alveolar capillary barrier in animal models of ALI [42]. The present data suggest that RAGE, TGF-*β*1, MMP2, and MMP9 may all participate in airway remodeling associated with inflammatory processes of the lung. During onset of ALI and ARDS, lung edema appears as a consequence of microvascular leakage associated with endothelial injury [43, 44].

Smad3 is considered the final integration factor of various fibrogenic signals and can regulate the expression of ECM-involved genes directly. Moreover, SIS3 is a small molecule which is a specific inhibitor of Smad3 and acts by inhibiting its phosphorylation. In this study, the lungs of the LPS-induced ARDS rats showed markedly increased levels of phosphorylated Smad3 when compared to the control group, and the signaling ligand, TGF-*β*1, also exhibited increased levels of expression. However, levels of phosphorylated Smad3 and TGF-*β*1 were both found to be suppressed in the SIS3-treated lungs of LPS-induced ARDS rats. These findings suggest that blocking Smad3 phosphorylation by SIS3 may have a therapeutic potential for ARDS, although further studies are required to confirm these findings.

TGF-*β* signaling is necessary in many fibrogenic events, including fibroblast activation and eventual ECM deposition [45–47]. SIS3 is a useful tool to evaluate TGF-*β*-regulated cellular mechanisms via selective inhibition of Smad3 [27]. The corresponding results suggest that SIS3 significantly inhibited LPS-induced ALI and significantly improved the lung histopathological features of LPS-induced ARDS in rats as well as reducing the expression levels of RAGE, TGF-*β*, MMP2, and MMP9. Recent studies have indicated that SIS3 treatment delays the early development of diabetic nephropathy in type I diabetic mouse models by inhibiting epithelial-mesenchymal transition and fibrosis [48].

SIS3 has also been shown to attenuate BLM-induced PF in mice [28]. SIS3 has been shown to protect unilateral ureteral obstruction of the kidneys against fibrosis, apoptosis, and inflammation injury through the inhibition of TGF-*β*/Smad3 signaling [49]. Through Smad-dependent signal transduction [50], TGF upregulates MMP2 and changes the matrix components to promote epithelial repair [51]. Another study carried out an immunohistochemical analysis and demonstrated that MMP2 and MMP9 expression was evident in alveolar macrophages and interstitial neutrophils [52]. The present study demonstrated through *in vivo* experiments that LPS increased RAGE, TGF-*β*1, MMP2, and MMP9 expression in the ECM and caused rat lung injury. Furthermore, this study illustrated that SIS3 inhibited these effects and reduced lung injury in ARDS rats. Immunohistochemical staining indicated that pretreatment of ARDS rats with SIS3 reduced lung injury caused by LPS, which was consistent with a previous study showing that SIS3 attenuated BLM-induced PF in mice [28]. Moreover, pharmacological inhibition of Smad3 or phospho-Smad3 has been shown to reduce BLM-induced PF in rats [53, 54]. The findings of this study indicated that blocking of Smad3 by the chemical inhibitor, SIS3, had a tendency to repair the ECM.

Intratracheal instillation of LPS induces acute lung inflammation with infiltration of inflammatory cells, which synthesize and secrete a wide variety of cytokines, chemokines, reactive oxygen species, and proteases, leading to aberrant fibroproliferation and matrix synthesis in mice. In this study, the intratracheal injection of LPS resulted in lung injury characterized by a significant increase in the lung *W*/*D* ratio and BALF total protein concentration, an upregulated inflammatory response with an increased number of total cells, an increased number of neutrophils, and an increased neutrophil ratio in the BALF. In addition, pretreatment with SIS3 significantly decreased the lung *W*/*D* ratio as an index of interstitial edema, number of total cells, neutrophils, and the neutrophil ratio in the BALF in LPS-induced rats. These findings were consistent with a previous study that demonstrated that the number of lymphocytes, macrophages, and neutrophils in the BALF was significantly decreased after administration of SIS3 to BLM-treated mice [28]. Overall, the present study showed that the inhibitory effect of SIS3 on LPS-induced ARDS in the rat model may be due to its inhibition of acute pulmonary inflammatory cell infiltration, regulation of cytokines, and reduction of related collagen synthesis.

## 5. Conclusions

The inhibitory effect of SIS3 on LPS-induced ALI in ARDS rats may be related to the inhibition of inflammatory cell infiltration, regulation of cytokine secretion, and degradation of collagen synthesis, thereby remodeling and repairing the ECM by TGF-*β*/Smad3. This may be a potential area for exploring a novel therapeutic strategy to combat ARDS.

## Figures and Tables

**Table 1:** The rat primer sequences used in this study.

Gene	GenBank number	Sense primer (5′-3′)	Antisense primer (5′-3′)
RAGE	G170807E03	ACCTTCAGGCTCAACCAAC	GGGACTCTTCACGCTTCG
TGF-*β*1	59086	ACCAAGGAGACGGAATACAGG	AGGTGTTGAGCCCTTTCCAG
MMP2	G170807E05	ACCACGGATCTGAGCAAT	TACTGGACCCACGCCTAC
MMP9	G170807E09	TTGGCTTCCTCCGTGATT	CCCTACTGCTGGTCCTTC
*β*-Actin	G170807E01	GAGAGGGAAATCGTGCGT	GGAGGAAGAGGATGCGC

## Data Availability

The datasets and codes generated or analyzed in this study are available from the corresponding author upon reasonable request.
